# Genome-wide association study using family-based cohorts identifies the *WLS* and *CCDC170/ESR1* loci as associated with bone mineral density

**DOI:** 10.1186/s12864-016-2481-0

**Published:** 2016-02-25

**Authors:** Benjamin H. Mullin, John P. Walsh, Hou-Feng Zheng, Suzanne J. Brown, Gabriela L. Surdulescu, Charles Curtis, Gerome Breen, Frank Dudbridge, J. Brent Richards, Tim D. Spector, Scott G. Wilson

**Affiliations:** Department of Endocrinology & Diabetes, Sir Charles Gairdner Hospital, Nedlands, Western Australia; School of Medicine and Pharmacology, University of Western Australia, Nedlands, Western Australia; Institute of Aging Research, School of Medicine, Hangzhou Normal University, and the Affiliated Hospital of Hangzhou Normal University, Hangzhou, Zhejiang China; Department of Twin Research & Genetic Epidemiology, King’s College London, London, UK; MRC Social, Genetic & Developmental Psychiatry Centre, Institute of Psychiatry, Psychology & Neuroscience, King’s College London, London, UK; NIHR Biomedical Research Centre for Mental Health, Maudsley Hospital and Institute of Psychiatry, Psychology & Neuroscience, King’s College London, London, UK; Department of Non-communicable Disease Epidemiology, London School of Hygiene and Tropical Medicine, London, UK; Departments of Medicine, Human Genetics, Epidemiology and Biostatistics, Jewish General Hospital, Lady Davis Institute, McGill University, Montreal, Canada

**Keywords:** Osteoporosis, BMD, GWAS, Polygenic, SNP, Pleiotropic

## Abstract

**Background:**

Osteoporosis is a common and debilitating bone disease that is characterised by a low bone mineral density (BMD), a highly heritable trait. Genome-wide association studies (GWAS) have proven to be very successful in identifying common genetic variants associated with BMD adjusted for age, gender and weight, however a large portion of the genetic variance for this trait remains unexplained. There is evidence to suggest significant genetic correlation between body size traits and BMD. It has also recently been suggested that unintended bias can be introduced as a result of adjusting a phenotype for a correlated trait. We performed a GWAS meta-analysis in two populations (total *n* = 6,696) using BMD data adjusted for only age and gender, in an attempt to identify genetic variants associated with BMD including those that may have potential pleiotropic effects on BMD and body size traits.

**Results:**

We observed a single variant, rs2566752, associated with spine BMD at the genome-wide significance level in the meta-analysis (*P = 3.36 × 10*^*−09*^). Logistic regression analysis also revealed an association between rs2566752 and fracture rate in one of our study cohorts (*P = 0.017*, *n* = 5,654). This is an intronic variant located in the *wntless Wnt ligand secretion mediator* (*WLS*) gene (1p31.3), a known BMD locus which encodes an integral component of the Wnt ligand secretion pathway. Bioinformatics analyses of variants in moderate LD with rs2566752 produced strong evidence for a regulatory role for the variants rs72670452, rs17130567 and rs1430738. Expression quantitative trait locus (eQTL) analysis suggested that the variants rs12568456 and rs17130567 are associated with expression of the *WLS* gene in whole blood, cerebellum and temporal cortex brain tissue (*P = 0.034–1.19 × 10*^*−23*^). Gene-wide association testing using the VErsatile Gene-based Association Study 2 (VEGAS2) software revealed associations between the *coiled-coil domain containing 170* (*CCDC170*) gene, located adjacent to the *oestrogen receptor 1* (*ESR1*) gene, and BMD at the spine, femoral neck and total hip sites (*P = 1.0 × 10*^*−06*^, *2.0 × 10*^*−06*^ and *2.0 × 10*^*−06*^ respectively).

**Conclusions:**

Genetic variation at the *WLS* and *CCDC170/ESR1* loci were found to be significantly associated with BMD adjusted for only age and gender at the genome-wide level in this meta-analysis.

## Background

Osteoporosis is a common and debilitating bone disease that is characterised by a low bone mineral density (BMD) and micro architectural deterioration of the bone tissue, leading to decreased bone strength and an increased risk of fracture [1]. Excess mortality caused by osteoporotic fracture in women has been estimated at 9 % 1-year post fracture and 24 % 5-years post fracture [2]. The disease is particularly prevalent in postmenopausal women due to a reduction in oestrogen production, with subsequent effects on bone as well as intestinal and renal calcium handling [3, 4]. Environmental factors, such as dietary calcium intake and exercise, also play a role in the disease [5, 6].

In addition to the effects of oestrogen and environmental factors, osteoporosis has a strong genetic component. BMD, as assessed by dual-energy X-ray absorptiometry (DXA), is currently the best clinical indicator of fracture risk and is a highly heritable trait. Twin and family studies have generated BMD heritability estimates ranging from 0.46–0.92 depending on the anatomical site studied [7, 8], while individuals with an affected first-degree relative have a considerably elevated familial relative risk of fragility fracture of 1.31–4.24 [9, 10]. Various measures of body size, including height and body mass index (BMI), have also been shown to have substantial heritable components [11, 12]. There is evidence to suggest significant genetic correlation between these traits and BMD [13, 14], and the existence of genes with pleiotropic effects [15].

Genome-wide association studies (GWAS) have proven to be very successful in identifying common genetic variants associated with BMD, with at least 71 loci reported as associated at a high level of confidence (NHGRI GWAS Catalogue [16]). However, a large portion of the genetic variance for BMD remains unexplained and many of the most significantly associated variants appear to contribute little to fracture risk [17]. The vast majority of genetic studies for BMD so far have used weight, age and gender as covariates to identify genetic variants associated with BMD independently of these factors. However, this potentially removes some of the influence of genes with pleiotropic effects on body size and BMD, and it has recently been suggested that unintended bias can be introduced as a result of adjusting a phenotype for a correlated trait [18]. We decided to perform a GWAS meta-analysis using BMD data adjusted for only age and gender, in an attempt to identify genetic variants associated with BMD including those that may have potential mediated pleiotropic effects on body size traits and bone density (whereby the skeleton adapts to the extra load by increasing bone density, or excess fat mass leads to altered secretion of bone active hormones such as oestrogen, leptin and adiponectin [19]), while also removing the possibility of false positives induced by collider bias.

## Methods

### Genetics of Osteoporosis (GENOS) cohort

The discovery population used in this study is known as the Genetics of Osteoporosis (GENOS) cohort. This cohort is based on ~1,050 individuals from an extreme discordant and concordant (EDAC) family-based study of Northern European/UK ancestry [20–22]. The EDAC families were selected based on containing a proband aged 25–83 years and having a lumbar spine, femoral neck or total hip BMD Z-score (defined as the number of standard deviations above or below the mean BMD of an age and gender matched control population) of < −1.5. The GENOS cohort is a powerful resource for detection of loci relevant to osteopenia and osteoporosis due to the EDAC study design and enrichment of genetic effects [23]. Clinical data were collected for BMD at the spine, total hip and femoral neck as well as extensive medical and lifestyle data. Exclusion criteria were applied and included hyperparathyroidism, long-term steroid use (>6 months), rheumatoid arthritis, anorexia nervosa or surgical oophorectomy. All subjects from the study provided written informed consent and the experimental protocols were approved by the Sir Charles Gairdner Group Human Research Ethics Committee and the St Thomas’ Hospital Research Ethics Committee.

At a clinic visit, data including age, height, weight, medical, gynaecological, and lifestyle factors were recorded and a blood sample was collected for DNA extraction. DXA BMD was assessed (Hologic, Bedford, MA, USA) at the lumbar spine L1–L4, femoral neck and total hip. BMD data were adjusted for age and gender prior to analysis by conversion to BMD Z-scores using the formula: (patient BMD – mean BMD of age and gender matched control population)/standard deviation. The NHANES (National Health and Nutrition Examination Survey) 3 reference ranges were used to calculate BMD Z-scores at the femoral neck and total hip sites, while the Hologic reference range was used for the lumbar spine.

### TwinsUK cohort

The replication population used in this study is known as the TwinsUK cohort and is comprised of ~12,000 monozygotic and dizygotic twins unselected for any particular disease or trait from St Thomas’ UK Adult Twin Registry (TwinsUK). The cohort is from Northern European/UK ancestry and has been shown to be representative of singleton populations and the UK population in general [24]. Medical and lifestyle-factor data were obtained from questionnaires, with exclusion criteria applied including rheumatoid arthritis, oral steroid use or surgical oophorectomy. All participants provided written, informed consent and the research was approved by the Guy’s and St Thomas’ Hospital Research Ethics Committee.

Clinical data for most of the twins were obtained at several time points for multiple phenotypes including fracture data (any fracture since 16 years of age) and DXA BMD (Hologic, Bedford, MA, USA) at the lumbar spine, femoral neck and total hip sites. DNA for genotyping was extracted from whole blood samples obtained for the vast majority of the cohort at the time of the study visit. BMD measures were adjusted for age and gender by conversion to BMD Z-scores as described above.

### Genotyping and imputation

Genotyping was performed for 1,046 individuals in the GENOS cohort using the Illumina HumanOmniExpress-12 v1.1 700 K BeadChip, with genotypes called using the GenCall algorithm (GenomeStudio). Quality control criteria were applied and included gender, ancestry (principal components analysis (EIGENSTRAT) [25]) and relatedness (Genome-wide Complex Trait Analysis (GCTA) [26]) checks. Samples with a genotype call rate <99 % were excluded as were any that were not of Northern European ancestry or that failed the above checks. Variants with a call rate <90 % or Hardy-Weinberg *P ≤ 10*^*−6*^ were also excluded. The genotype data was pre-phased using SHAPEIT2 [27] before imputation was performed using the IMPUTE2 software package [28] in conjunction with the 1000 Genomes Project Phase 1 and UK10K Project reference panels. Imputed variants with an “info” score <0.4 were excluded.

Genotyping in the TwinsUK cohort was completed for 5,654 individuals using the Illumina HumanHap300, HumanHap610, 1 M-Duo and 1.2 M-Duo arrays, as described previously [29, 30]. Imputation was performed using IMPUTE2 [28] in conjunction with the 1000 Genomes Project Phase 1 reference panel. Imputed variants with an info score <0.4 were excluded.

### Statistical analysis

Genome-wide association analyses for BMD at the lumbar spine, femoral neck and total hip sites was performed in each cohort using GEMMA (Genome-wide Efficient Mixed Model Association) [31], which controls for familial relatedness within a cohort. Only variants with a minor allele frequency (MAF) ≥1 % were included in the analysis. Meta-analysis of the results from the two cohorts was performed using the GWAMA (Genome-Wide Association Meta-Analysis) software package [32] – a fixed effects model was applied, combining estimates of the allelic effect size and standard error from each cohort. Genome-wide significant and suggestive thresholds were set at *1.17 × 10*^*−08*^ [33, 34] and *5 × 10*^*−07*^ respectively. Assuming an additive genetic model, our combined cohorts (total *n* = 6,696) have an estimated 88.7 % power to detect a variant with a MAF of 1 % that accounts for 0.2 % of the trait variance [35]. Any variants associated with BMD at the genome-wide significance level were tested for association with fracture rate in the TwinsUK cohort using logistic regression adjusted for age, age^2^, gender, height and weight (SPSS Statistics 22). Gene-wide (+/− 10Kb) tests of association with BMD were performed on the GWAS meta-analysis results using the VErsatile Gene-based Association Study 2 (VEGAS2) software [36], which assigns variants to genes and calculates gene-based empirical association p-values while accounting for the LD structure within the gene. We have found the +/− 10 Kb option to be a good balance between incorporating short-range regulatory variants while maintaining the specificity of the result for a specific gene, as variants associated with neighbouring genes can influence the test statistic for a gene of interest. A genome-wide Bonferroni-corrected significance threshold of *2.14 × 10*^*−06*^ for 23,390 gene tests was used, with suggestive significance set at *1 × 10*^*−05*^.

### Bioinformatics analysis

Analysis of the linkage disequilibrium (LD) surrounding variants of interest was performed using LDlink (1000 Genomes Project Phase 3 EUR population) [37]. Prediction of histone marks, DNAse hypersensitivity sites and expression quantitative trait locus (eQTL) associations was performed using HaploReg v4.0 [38] and genomic evolutionary rate profiling (GERP) scores were obtained using GWAVA (Genome Wide Annotation of Variants) [39]. Variants of interest were also queried using the human osteoblast eQTL dataset generated by Grundberg et al. [40].

## Results

**Fig. 1:**
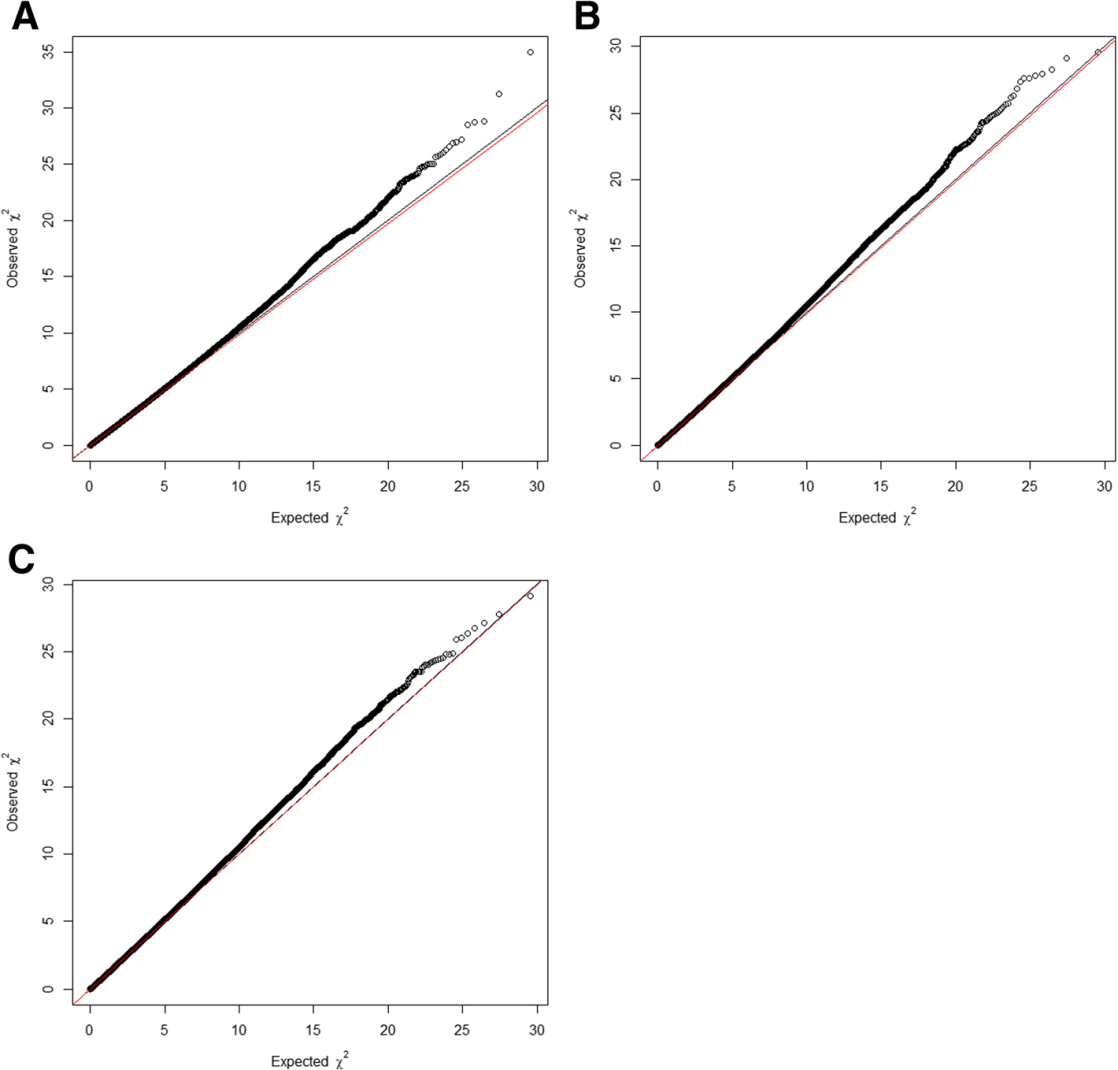
Quantile-quantile plots for (**a**) spine, (**b**) femoral neck and (**c**) total hip BMD. Plots display observed p values versus expected p values from the meta-analysis results

Descriptive statistics for the two cohorts are presented in Table 1 and quantile-quantile plots generated for the meta-analysis results are shown in Fig. 1a, b and c (spine BMD λ = 0.99, femoral neck BMD λ = 0.99, total hip BMD λ = 1.00). Genome-wide association analysis of the family-based discovery cohort (GENOS), enriched for individuals with low BMD, did not yield any genome-wide significant results. Therefore, we next performed a meta-analysis of both discovery and replication cohorts.

**Table 1:** Descriptive statistics for the two cohorts used in the study

	GENOS	TwinsUK
Subjects (N)	1,042	5,654
Age, mean (SD)	55.236 (11.658)	47.001 (12.647)
Age (range)	20–83	16–81
Male (%)	1.9	8.3
Spine BMD (g/cm^2^), mean (SD)	0.878 (0.158)	1.002 (0.143)
Spine BMD Z, mean (SD)	−0.446 (1.305)	0.230 (1.267)
Total hip BMD (g/cm^2^), mean (SD)	0.813 (0.141)	0.941 (0.133)
Total hip BMD Z, mean (SD)	−0.319 (1.058)	0.377 (0.992)
Femoral neck BMD (g/cm^2^), mean (SD)	0.723 (0.139)	0.816 (0.129)
Femoral neck BMD Z, mean (SD)	−0.123 (1.147)	0.319 (1.049)
Fracture rate (%)	NA	28.3

### Spine BMD

**Fig. 2:**
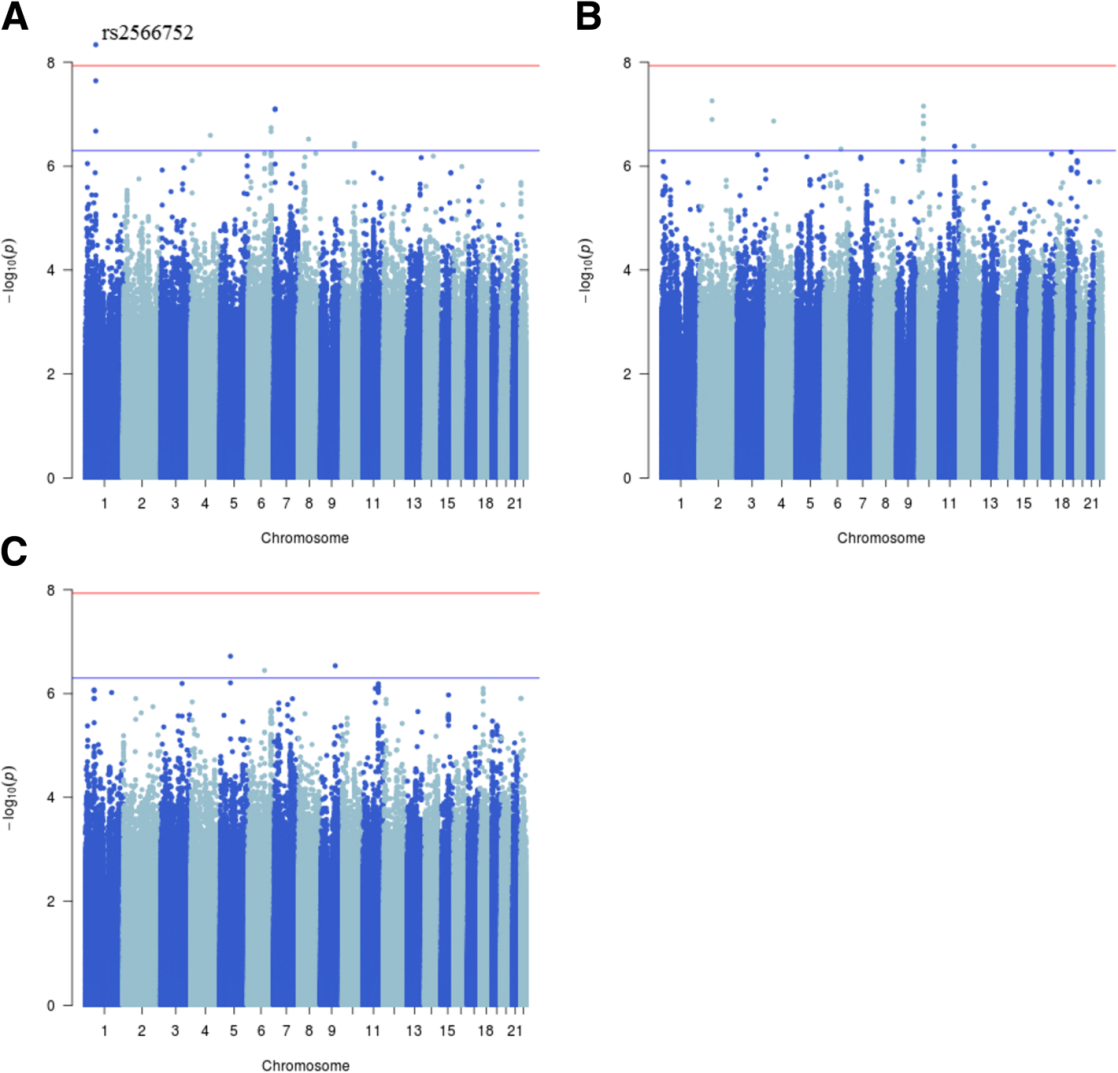
Manhattan plots for (**a**) spine, (**b**) femoral neck and (**c**) total hip BMD. Each plot depicts the variants genotyped across the 22 autosomes against the –log_10_ *P* value from the meta-analysis. The blue line represents the suggestive significance threshold of *5 × 10* ^*−07*^ and the red line represents the genome-wide significance threshold of *1.17 × 10* ^*−08*^

**Fig. 3:**
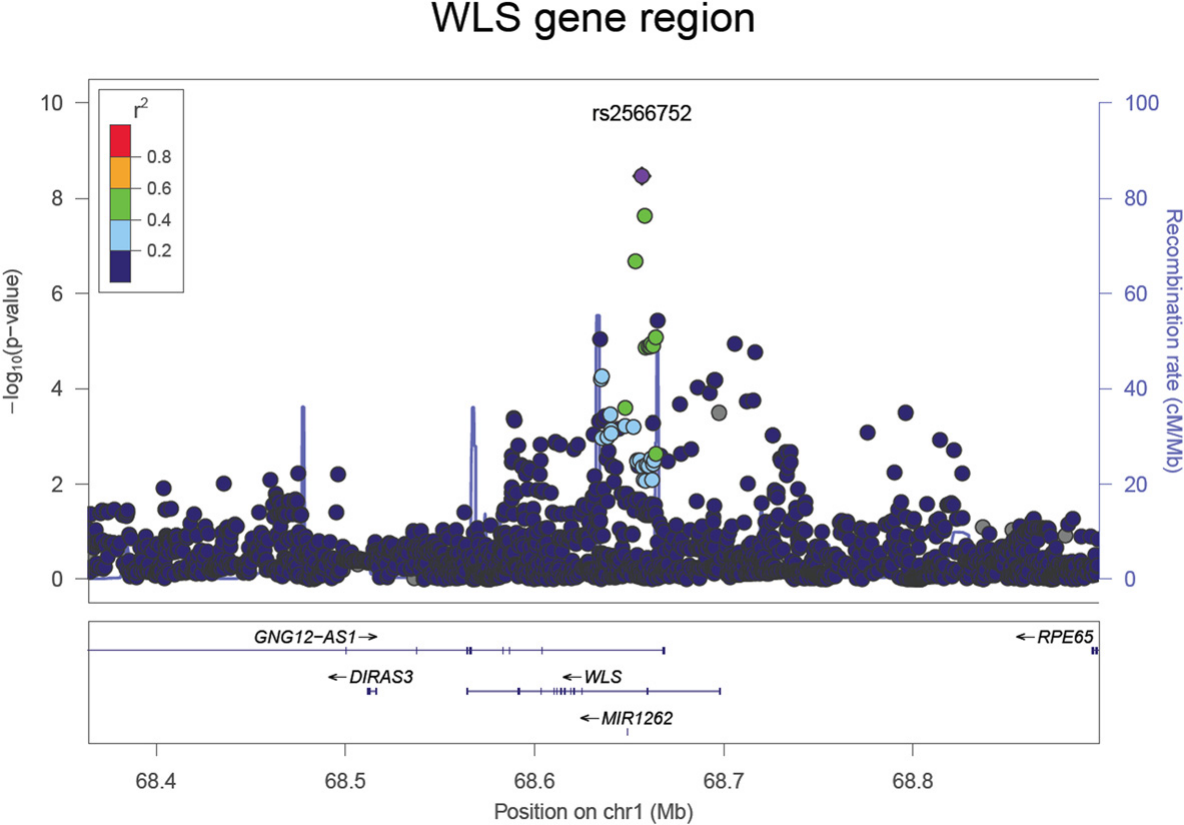
Regional association plot for spine BMD from the *WLS* gene region. Genetic variants within 200 kb of the *WLS* gene are depicted (x axis) along with their meta-analysis *P* value (−log_10_) from the spine BMD analysis. Variants are colour coded according to their LD (r^2^) with the lead SNP (1000 Genomes Project Nov 2014 EUR population). The recombination rate (blue line) and position of genes, their exons and direction of transcription is also indicated [69]

We observed a single variant, rs2566752, associated with spine BMD at the genome-wide significance level in the meta-analysis (*P = 3.36 × 10*^*−09*^) (Fig. 2a) (Table 2). This is an intronic variant (*C/T*) located in the *wntless Wnt ligand secretion mediator* (*WLS*) gene (1p31.3) (Fig. 3), the less common *C* allele being associated with an increased spine BMD. This allele was also found to be associated with a decreased risk of fracture in the TwinsUK cohort (odds ratio (OR) = 0.86 (95 % confidence interval (CI): 0.77–0.97), *P = 0.017*).

**Table 2:** Genome-wide significant and suggestive loci identified in the meta-analysis

						GENOS (*n* = 1,042)		TwinsUK (*n* = 5,654)		Meta-analysis (*n* = 6,696)			
Phenotype	Locus	rsID	EA	OA	EAF	Beta	P	Beta	P	Beta	SE	*P* value	Nearby genes
Spine BMD	1p31.3	rs2566752	C	T	0.38	0.28	2.3E-5	0.14	1.3E-6	0.16	0.03	3.4E-9	*WLS*
	4q28.1	rs4423886	C	T	0.19	−0.17	0.07	−0.22	4.6E-7	−0.21	0.04	2.5E-7	*FAT4*
	6q25.1	rs1038304	G	A	0.51	−0.21	8.3E-4	−0.12	1.8E-5	−0.13	0.03	1.8E-7	*CCDC170*/*ESR1*
	7p22.1	rs188966536	T	G	0.02	1.21	1.8E-6	0.51	1.6E-4	0.67	0.12	7.8E-8	*CCZ1B*
	8q13.1	rs76115211	A	T	0.07	−0.30	0.04	−0.35	7.9E-7	−0.34	0.07	3.0E-7	*LINC00251*
	10q22.3	rs114936111	A	G	0.22	0.29	3.0E-4	0.14	4.1E-5	0.16	0.03	3.6E-7	*KCNMA1*
Femoral Neck BMD	2p12	rs12998155	C	T	0.45	−0.15	6.7E-3	−0.13	8.0E-7	−0.13	0.02	5.4E-8	*CTNNA2*
	4p13	rs17885864	T	G	0.03	−0.56	1.6E-3	−0.35	4.2E-6	−0.38	0.07	1.3E-7	*PHOX2B*
	6q21	rs117359272	G	C	0.02	0.55	5.8E-3	0.38	1.6E-5	0.41	0.08	4.6E-7	*LINC00222*
	10p11.23	rs73245065	G	C	0.02	−0.70	1.0E-4	−0.32	1.4E-5	−0.37	0.07	6.9E-8	*LYZL2*
	11q22.1	rs10893396	C	G	0.17	0.17	0.02	0.14	5.9E-6	0.15	0.03	4.1E-7	*CNTN5*
	12q21.33	rs191780267	C	T	0.01	0.69	0.02	0.57	4.7E-6	0.59	0.11	4.1E-7	*LOC728084*
Total Hip BMD	5q13.1	rs78935958	G	T	0.06	−0.38	2.8E-3	−0.24	6.1E-6	−0.26	0.05	1.9E-7	*LOC101928858*/*PIK3R1*
	6q21	rs117359272	G	C	0.02	0.56	1.9E-3	0.35	2.9E-5	0.39	0.08	3.6E-7	*LINC00222*
	9q22.31	rs1907805	T	C	0.04	0.21	0.18	0.33	2.5E-7	0.31	0.06	2.9E-7	*ROR2*

Only the maximally associated variant from each locus is shown. *EA* effect allele, *OA* other allele, *EAF* effect allele frequency, *SE* standard error

Five additional loci demonstrated genome-wide suggestive associations (*P < 5 × 10*^*−07*^) with spine BMD, including 4q28.1 (*FAT4*), 6q25.1 (*CCDC170*/*ESR1*), 7p22.1 (*CCZ1B*), 8q13.1 (*LINC00251*) and 10q22.3 (*KCNMA1*) (Table 2).

### Femoral neck BMD

No variants were found to be associated with femoral neck BMD at the genome-wide significance level in the meta-analysis (Fig. 2b). However, genome-wide suggestive associations were seen at 6 loci, including 2p12 (*CTNNA2*), 4p13 (*PHOX2B*), 6q21 (*LINC00222*), 10p11.23 (*LYZL2*), 11q22.1 (*CNTN5*) and 12q21.33 (*LOC728084*) (Table 2).

### Total hip BMD

No variants were found to be associated with total hip BMD at the genome-wide significance level in the meta-analysis (Fig. 2c). However, genome-wide suggestive associations were seen at 3 loci, including 5q13.1 (*LOC101928858*/*PIK3R1*), 6q21 (*LINC00222*) and 9q22.31 (*ROR2*) (Table 2).

### Gene-wide association testing

**Fig. 4:**
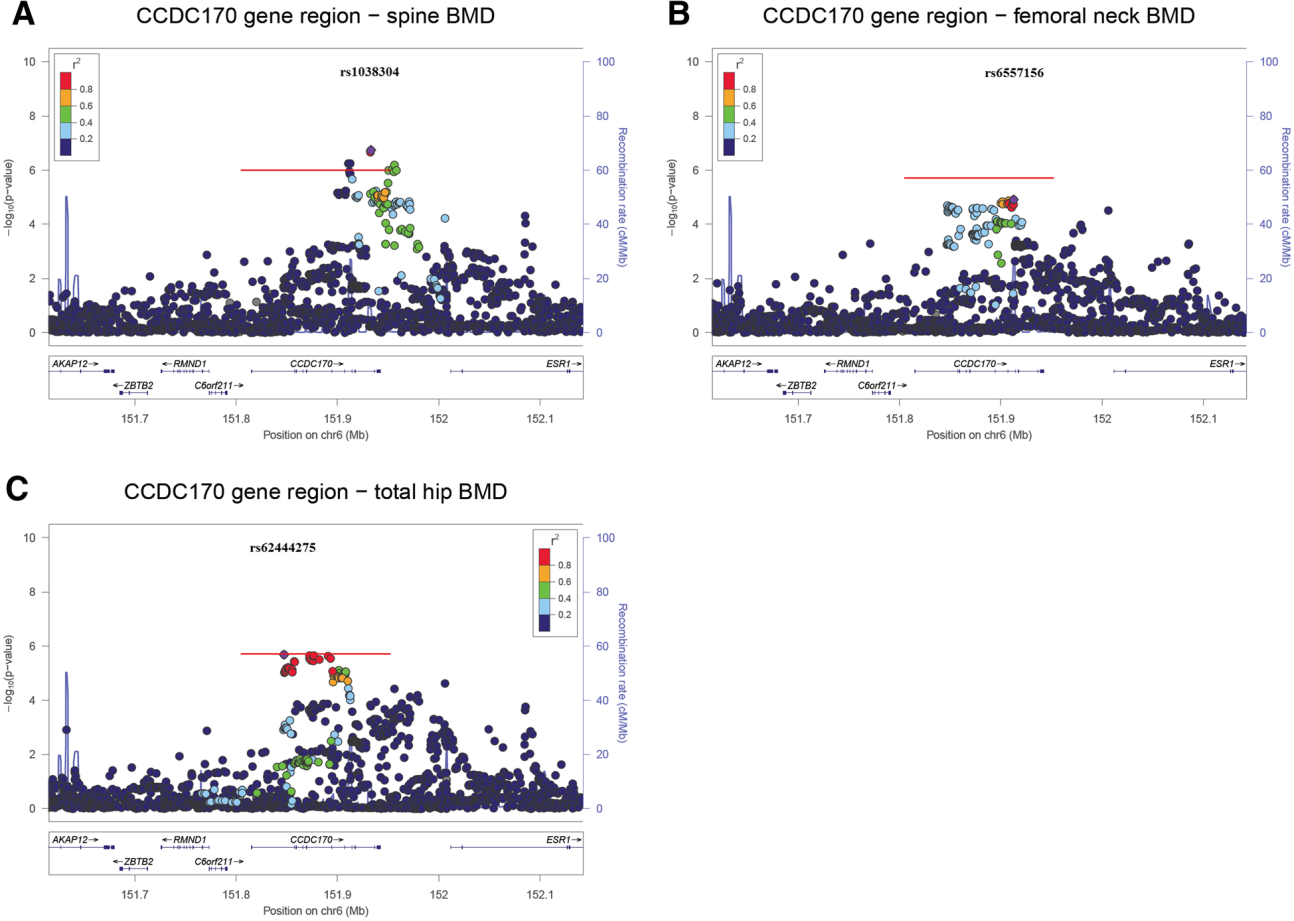
Regional association plots for the *CCDC170* locus for (**a**) spine, (**b**) femoral neck and (**c**) total hip BMD. Genetic variants within 200 kb of the *CCDC170* gene are depicted (x axis) along with their meta-analysis *P* value (−log_10_). Variants are colour coded according to their LD (r^2^) with the lead SNP (1000 Genomes Project Nov 2014 EUR population). The red bars represent the gene-wide region tested and association *P* value (−log_10_). The recombination rate (blue line) and position of genes, their exons and direction of transcription is also indicated [69]

The VEGAS2 software identified the *CCDC170* gene as associated with spine, femoral neck and total hip BMD at the genome-wide significance level (*P = 1.0 × 10*^*−06*^, *2.0 × 10*^*−06*^ and *2.0 × 10*^*−06*^ respectively). The maximally associated variants for each phenotype were rs1038304 (spine, *P = 1.8 × 10*^*−07*^), rs6557156 (femoral neck, *P = 1.23 × 10*^*−5*^) and rs62444275 (total hip, *P = 2.08 × 10*^*−6*^) (Fig. 4). No other genes were found to be associated with any of the BMD phenotypes at the genome-wide significance level. Suggestive associations were seen for spine BMD with the genes *HOXC5* and *HOXC6*, which are located in close proximity on chromosome 12 (*P = 1.0 × 10*^*−05*^ and *1.0 × 10*^*−05*^ respectively). No other suggestive associations were seen.

### Bioinformatics analysis

Analysis of LD in the *WLS* gene region showed there were no variants in strong LD (r^2^ > 0.8) and 10 variants in moderate LD (r^2^ > 0.5) with rs2566752 (Table 3). All of these variants were associated with spine BMD *P < 5 × 10*^*−05*^ in the meta-analysis, apart from rs1430738 (*P = 0.002*) and rs36009202 (not present in genotype dataset). Bioinformatics analysis of these variants suggested the presence of various regulatory features including promoter histone marks, enhancer histone marks and DNAse hypersensitivity sites in multiple cell types for the variants rs72670452, rs17130567 and rs1430738 (Table 3). According to the HaploReg v4.0 eQTL database [38], which contains eQTL data for a variety of tissues including results from the pilot phase of the Genotype-Tissue Expression (GTEx) project [41], the two variants rs12568456 and rs17130567 were also associated with expression of the *WLS* gene in whole blood, cerebellum and temporal cortex brain tissue (*P = 0.034–1.19 × 10*^*−23*^) [42, 43]. In all three tissues, the less common allele at these two variants was associated with increased expression of the *WLS* gene. The variant rs72670452 was found to have a GERP score of 3.95 (Table 3), suggesting that the site may be under evolutionary constraint. GERP scores are used to quantify nucleotide substitution deficits, which represent “rejected substitutions” that reflect past purifying selection [44]. Apart from rs36009202 and rs75334237, which were not present in the dataset, none of the variants in Table 3 were found to be associated with expression of the *WLS* gene in primary human osteoblasts (*P = 0.16–0.92*) [40].

**Table 3:** Bioinformatics analysis of variants in moderate LD (r^2^ > 0.5) with rs2566752

Variant	Relative position^a^	LD with rs2566752 (r^2^)	GERP score	Promoter histone marks	Enhancer histone marks	DNAse	eQTL results
rs2026749	−3208	0.54	1.26	-	-	-	ND
rs2566752	0	1	−0.317	-	-	-	ND
rs36009202	+112	0.57	0.697	-	-	-	ND
rs75334237 (rs5774922)	+1570	0.53	0	-	-	-	ND
rs2033345	+2060	0.52	0.352	-	-	-	ND
rs2033344	+3701	0.51	−2.5	-	1 tissue	-	ND
rs57748040	+4196	0.51	−3.48	-	1 tissue	-	ND
rs12568456	+4789	0.51	−5.1	-	2 tissues	-	3 studies
rs72670452	+6143	0.51	3.95	5 tissues	12 tissues	5 tissues	ND
rs17130567	+7150	0.51	0.805	4 tissues	18 tissues	12 tissues	3 studies
rs1430738	+7432	0.6	−0.599	2 tissues	17 tissues	-	ND

^a^Relative to rs2566752 (hg19); GERP, genomic evolutionary rate profiling; eQTL, expression quantitative trait locus; ND, no data

Analysis of the LD between the maximally associated variants from the *CCDC170* gene for each BMD phenotype suggested that the rs6557156 and rs62444275 variants are in moderate LD (r^2^ = 0.31), whereas the rs1038304 variant is in low LD with rs6557156 and rs62444275 (r^2^ = 0.05 and 0.02 respectively).

## Discussion

We undertook this study to identify genes significantly associated with BMD adjusted for only age and gender in order to investigate all genes regulating BMD including those with possible pleiotropic effects extending to aspects of body size. We have demonstrated a genome-wide significant association between a variant in the *WLS* gene (also known as *GPR177*) and BMD at the spine in this meta-analysis, as well as an association with fracture rate in the TwinsUK cohort. The product of the *WLS* gene is a chaperone protein that is an integral component of the Wnt ligand secretion pathway. There are 19 Wnt proteins in the mammalian genome, and they represent an evolutionarily conserved family of secreted signalling molecules. Both the canonical (β-catenin dependent) and non-canonical (β-catenin independent) Wnt signalling pathways have been shown to play important roles in prenatal and postnatal bone development [45]. The product of the *WLS* gene has been shown to be required for the activity of virtually all of the Wnt proteins [46] and *Wls*-knockout mice display early embryonic lethality due to impaired body axis formation [47]. Conditional deletion of the *Wls* gene in mice has been found to severely impair the development of the craniofacial and body skeletons, demonstrating a role in intramembranous and endochondral ossification respectively [48].

Genetic variation within the *WLS* gene has been identified as associated with BMD in several previous GWAS [17, 49–51], some of which have observed more than one independent association signal originating from the locus [17, 51]. The most recent of these studies, a large meta-analysis that included a subset of the TwinsUK cohort and used a combination of whole-genome sequence, whole-exome sequence and deeply imputed genotype data in its discovery study (n ≈ 33,000), also identified the rs2566752 single nucleotide polymorphism (SNP) as the maximally associated variant from the *WLS* gene region for both lumbar spine and femoral neck BMD [51]. Consistent with our findings, this variant was most strongly associated with BMD at the spine and the *C* allele was associated with an increased BMD [51]. Interestingly, pleiotropic effects on BMD and bone geometric parameters have been previously observed at the *WLS* locus [52], although it should be noted that to our knowledge variation in the *WLS* gene has not been previously associated with body size related traits such as height, weight or BMI. Liu et al. [15] published the results from a bivariate GWAS for BMI and hip BMD, identifying significant associations for two SNPs in the *SOX6* gene in males. No significant associations were seen for variants in the *WLS* locus, however only 380,000 variants were tested genome-wide and spine BMD was not analysed [15].

It would appear that the associations seen between rs2566752 and BMD are likely mediated through regulatory effects on the *WLS* gene. The bioinformatics analysis produced strong evidence for a regulatory role for the variants rs72670452, rs17130567 and rs1430738, which are in moderate LD with rs2566752. It should be noted, however, that there are limitations to using histone modification data when identifying regulatory elements and further study is required. eQTL data from the HaploReg v4.0 eQTL database were only available for variants in the HapMap2 variant set, however this data suggested that the variants rs12568456 and rs17130567 are significantly associated with expression of the *WLS* gene, with the less common allele at each variant associated with an increase in expression. Judging from the findings in mice described above, increased expression of *WLS* would likely lead to increased BMD, consistent with the observations for the less common alleles at these variants in this study. These eQTL findings should be interpreted with caution, however, as none of the *WLS* variants of interest were found to be associated with expression of the *WLS* gene in primary human osteoblasts derived from 95 donors [40].

Numerous genome-wide suggestive associations were seen for individual variants in this study, representing a mix of novel and known bone loci. Some of the more well-replicated BMD loci include 6q25.1 (*CCDC170*/*ESR1*) [17, 49, 51, 53, 54] and 10q22.3 (*KCNMA1*) [17, 51]. An interesting novel suggestive association was seen for spine BMD in the vicinity of the *FAT atypical cadherin 4* (*FAT4*) gene. Mutations in this gene have been shown to cause Van Maldergem Syndrome 2 [55], which is characterised by intellectual disability, characteristic craniofacial features and skeletal and limb malformations. Additional evidence for a role for this gene in bone comes from *Fat4*-deficient mice, which are born runted with a curved body axis, curved tail, abnormal vertebrae morphology and abnormal sternum ossification [56, 57]. Another interesting suggestive association was seen between femoral neck BMD and a variant in the *catenin (cadherin-associated protein), alpha 2* (*CTNNA2*) gene, which encodes an α-catenin that appears to have a role as a cell-cell adhesion protein by promoting the linking of β-catenin to the cytoskeleton and inhibiting its downstream signalling [58]. Genome-wide suggestive associations have been previously identified between variation in this gene and forearm BMD [59], while mice carrying a mutation affecting the *Ctnna2* gene weigh 25–50 % less than littermate controls [60], which suggests a potential role in growth or body size regulation.

Gene-wide association testing demonstrated associations between the *coiled-coil domain containing 170* (*CCDC170*) gene and BMD at the spine, femoral neck and total hip sites. The fact that this gene was identified as genome-wide significant by the VEGAS2 software, which corrects for LD within a gene, is likely indicative of the presence of multiple independent association signals for BMD originating from this locus. This phenomenon has previously been reported at this locus for bone phenotypes [17, 61, 62] and is supported by the fact that the maximally associated variant from the *CCDC170* gene region for each phenotype in this study were not in strong LD with each other. Gene-wide associations with spine and femoral neck BMD have been previously reported for this locus using HapMap Phase II LD data [63]. The *CCDC170* gene encodes a protein with unknown function and is located adjacent to the *ESR1* gene. The *ESR1* gene encodes the oestrogen receptor 1, a DNA-binding transcription factor that regulates the expression of many different genes. Oestrogen has a well-established protective effect on the skeleton by slowing the rate of bone remodelling and resorption while maintaining the rate of bone formation [64]. *Esr1*-knockout mice display decreased longitudinal bone growth, increased body weight [65] and obesity [66]. Loss of function mutations in the human *ESR1* gene result in a variety of skeletal phenotypes including tall stature, reduced BMD and cortical thinning, as well as impaired glucose tolerance and hyperinsulinemia [67, 68]. It is possible that the associations seen in this study between the *CCDC170* gene and BMD reflect the presence of regulatory elements relevant to the *ESR1* gene.

## Conclusions

In conclusion, we performed a GWAS for BMD adjusted for only age and gender using two family-based cohorts. The size of each cohort was limited in comparison to current standards for well-powered GWAS, and we were not able to detect any genome-wide significant loci using the discovery GENOS cohort alone. By conducting a meta-analysis of the two family-based cohorts, we confirmed that genetic variation at the *WLS* locus is significantly associated with BMD at the genome-wide level. Bioinformatics and eQTL analyses suggest that the association seen is likely caused by regulatory effects on the *WLS* gene. Gene-wide association testing revealed associations between the *CCDC170* gene and BMD at each site studied, although these associations may be due to the presence of regulatory elements relevant to the adjacent *ESR1* gene.

## Abbreviations

BMD: bone mineral density
BMI: body mass index
DXA: dual-energy X-ray absorptiometry
EDAC: extreme discordant and concordant
eQTL: expression quantitative trait locus
GEMMA: Genome-wide Efficient Mixed Model Association
GERP: genomic evolutionary rate profiling
GWAS: genome-wide association study
GWAVA: Genome Wide Annotation of Variants
LD: linkage disequilibrium
NHANES: National Health and Nutrition Examination Survey
VEGAS2: VErsatile Gene-based Association Study 2

## References

[1] Kanis JA, Melton LJ, Christiansen C, Johnston CC, Khaltaev N (1994). The diagnosis of osteoporosis. J Bone Miner Res.

[2] Robbins JA, Biggs ML, Cauley J (2006). Adjusted mortality after hip fracture: from the cardiovascular health study. J Am Geriatr Soc.

[3] Heaney RP, Recker RR, Stegman MR, Moy AJ (1989). Calcium absorption in women: relationships to calcium intake, estrogen status, and age. J Bone Miner Res.

[4] Nordin BE, Need AG, Morris HA, Horowitz M, Robertson WG (1991). Evidence for a renal calcium leak in postmenopausal women. J Clin Endocrinol Metab.

[5] Specker BL (1996). Evidence for an interaction between calcium intake and physical activity on changes in bone mineral density. J Bone Miner Res.

[6] Uusi-Rasi K, Sievanen H, Vuori I, Pasanen M, Heinonen A, Oja P (1998). Associations of physical activity and calcium intake with bone mass and size in healthy women at different ages. J Bone Miner Res.

[7] Pocock NA, Eisman JA, Hopper JL, Yeates MG, Sambrook PN, Eberl S (1987). Genetic determinants of bone mass in adults. A twin study. J Clin Invest.

[8] Krall EA, Dawson-Hughes B (1993). Heritable and life-style determinants of bone mineral density. J Bone Miner Res.

[9] Deng HW, Chen WM, Recker S, Stegman MR, Li JL, Davies KM, Zhou Y, Deng H, Heaney R, Recker RR. Genetic determination of Colles’ fracture and differential bone mass in women with and without Colles’ fracture. J Bone Miner Res. 2000;15(7):1243–52. 10.1359/jbmr.2000.15.7.1243 10893672

[10] Keen RW, Hart DJ, Arden NK, Doyle DV, Spector TD (1999). Family history of appendicular fracture and risk of osteoporosis: a population-based study. Osteoporos Int.

[11] Hemani G, Yang J, Vinkhuyzen A, Powell JE, Willemsen G, Hottenga JJ, Abdellaoui A, Mangino M, Valdes AM, Medland SE (2013). Inference of the genetic architecture underlying BMI and height with the use of 20,240 sibling pairs. Am J Hum Genet.

[12] Silventoinen K, Magnusson PK, Tynelius P, Kaprio J, Rasmussen F (2008). Heritability of body size and muscle strength in young adulthood: a study of one million Swedish men. Genet Epidemiol.

[13] Deng FY, Lei SF, Li MX, Jiang C, Dvornyk V, Deng HW (2006). Genetic determination and correlation of body mass index and bone mineral density at the spine and hip in Chinese Han ethnicity. Osteoporos Int.

[14] Yang YJ, Dvornyk V, Jian WX, Xiao SM, Deng HW (2005). Genetic and environmental correlations between bone phenotypes and anthropometric indices in Chinese. Osteoporos Int.

[15] Liu YZ, Pei YF, Liu JF, Yang F, Guo Y, Zhang L, Liu XG, Yan H, Wang L, Zhang YP (2009). Powerful bivariate genome-wide association analyses suggest the SOX6 gene influencing both obesity and osteoporosis phenotypes in males. PLoS One.

[16] Welter D, MacArthur J, Morales J, Burdett T, Hall P, Junkins H, Klemm A, Flicek P, Manolio T, Hindorff L (2014). The NHGRI GWAS Catalog, a curated resource of SNP-trait associations. Nucleic Acids Res.

[17] Estrada K, Styrkarsdottir U, Evangelou E, Hsu YH, Duncan EL, Ntzani EE, Oei L, Albagha OM, Amin N, Kemp JP (2012). Genome-wide meta-analysis identifies 56 bone mineral density loci and reveals 14 loci associated with risk of fracture. Nat Genet.

[18] Aschard H, Vilhjalmsson BJ, Joshi AD, Price AL, Kraft P (2015). Adjusting for heritable covariates can bias effect estimates in genome-wide association studies. Am J Hum Genet.

[19] Aguirre L, Napoli N, Waters D, Qualls C, Villareal DT, Armamento-Villareal R (2014). Increasing adiposity is associated with higher adipokine levels and lower bone mineral density in obese older adults. J Clin Endocrinol Metab.

[20] Mullin BH, Prince RL, Dick IM, Hart DJ, Spector TD, Dudbridge F, Wilson SG. Identification of a role for the ARHGEF3 gene in postmenopausal osteoporosis. Am J Hum Genet. 2008;82(6):1262–9. 10.1016/j.ajhg.2008.04.016 PMC2427258 18499081

[21] Mullin BH, Prince RL, Mamotte C, Spector TD, Hart DJ, Dudbridge F, Wilson SG. Further genetic evidence suggesting a role for the RhoGTPase-RhoGEF pathway in osteoporosis. Bone. 2009;45(2):387–91. 10.1016/j.bone.2009.04.254 19427924

[22] Wilson SG, Reed PW, Bansal A, Chiano M, Lindersson M, Langdown M, Prince RL, Thompson D, Thompson E, Bailey M (2003). Comparison of genome screens for two independent cohorts provides replication of suggestive linkage of bone mineral density to 3p21 and 1p36. Am J Hum Genet.

[23] Li M, Boehnke M, Abecasis GR (2006). Efficient study designs for test of genetic association using sibship data and unrelated cases and controls. Am J Hum Genet.

[24] Spector TD, Williams FM (2006). The UK adult twin registry (TwinsUK). Twin Res Hum Genet.

[25] Price AL, Patterson NJ, Plenge RM, Weinblatt ME, Shadick NA, Reich D (2006). Principal components analysis corrects for stratification in genome-wide association studies. Nat Genet.

[26] Yang J, Lee SH, Goddard ME, Visscher PM (2011). GCTA: a tool for genome-wide complex trait analysis. Am J Hum Genet.

[27] Delaneau O, Marchini J, Zagury JF (2012). A linear complexity phasing method for thousands of genomes. Nat Methods.

[28] Howie B, Fuchsberger C, Stephens M, Marchini J, Abecasis GR (2012). Fast and accurate genotype imputation in genome-wide association studies through pre-phasing. Nat Genet.

[29] Metrustry SJ, Edwards MH, Medland SE, Holloway JW, Montgomery GW, Martin NG, Spector TD, Cooper C, Valdes AM . Variants close to NTRK2 gene are associated with birth weight in female twins. Twin Res Hum Genet. 2014;17(4):254–61. 10.1017/thg.2014.34 24950379

[30] Richards JB, Rivadeneira F, Inouye M, Pastinen TM, Soranzo N, Wilson SG, Andrew T, Falchi M, Gwilliam R, Ahmadi KR (2008). Bone mineral density, osteoporosis, and osteoporotic fractures: a genome-wide association study. Lancet.

[31] Zhou X, Stephens M (2012). Genome-wide efficient mixed-model analysis for association studies. Nat Genet.

[32] Magi R, Morris AP (2010). GWAMA: software for genome-wide association meta-analysis. BMC Bioinformatics.

[33] Xu C, Tachmazidou I, Walter K, Ciampi A, Zeggini E, Greenwood CM (2014). Estimating genome-wide significance for whole-genome sequencing studies. Genet Epidemiol.

[34] Taylor PN, Porcu E, Chew S, Campbell PJ, Traglia M, Brown SJ, Mullin BH, Shihab HA, Min J, Walter K (2015). Whole-genome sequence-based analysis of thyroid function. Nat Commun.

[35] Purcell S, Cherny SS, Sham PC (2003). Genetic power calculator: design of linkage and association genetic mapping studies of complex traits. Bioinformatics.

[36] Mishra A, Macgregor S (2015). VEGAS2: software for more flexible gene-based testing. Twin Res Hum Genet.

[37] Machiela MJ, Chanock SJ (2015). LDlink: a web-based application for exploring population-specific haplotype structure and linking correlated alleles of possible functional variants. Bioinformatics.

[38] Ward LD, Kellis M (2012). HaploReg: a resource for exploring chromatin states, conservation, and regulatory motif alterations within sets of genetically linked variants. Nucleic Acids Res.

[39] Ritchie GR, Dunham I, Zeggini E, Flicek P (2014). Functional annotation of noncoding sequence variants. Nat Methods.

[40] Grundberg E, Kwan T, Ge B, Lam KC, Koka V, Kindmark A, Mallmin H, Dias J, Verlaan DJ, Ouimet M (2009). Population genomics in a disease targeted primary cell model. Genome Res.

[41] Human genomics (2015). The Genotype-Tissue Expression (GTEx) pilot analysis: multitissue gene regulation in humans. Science.

[42] Westra HJ, Peters MJ, Esko T, Yaghootkar H, Schurmann C, Kettunen J, Christiansen MW, Fairfax BP, Schramm K, Powell JE (2013). Systematic identification of trans eQTLs as putative drivers of known disease associations. Nat Genet.

[43] Zou F, Chai HS, Younkin CS, Allen M, Crook J, Pankratz VS, Carrasquillo MM, Rowley CN, Nair AA, Middha S (2012). Brain expression genome-wide association study (eGWAS) identifies human disease-associated variants. PLoS Genet.

[44] Cooper GM, Stone EA, Asimenos G, Green ED, Batzoglou S, Sidow A (2005). Distribution and intensity of constraint in mammalian genomic sequence. Genome Res.

[45] Zhong ZA, Zahatnansky J, Snider J, Van Wieren E, Diegel CR, Williams BO. Wntless spatially regulates bone development through beta-catenin-dependent and independent mechanisms. Dev Dyn. 2015. 10.1002/dvdy.24316 PMC4844555 26249818

[46] Najdi R, Proffitt K, Sprowl S, Kaur S, Yu J, Covey TM, Virshup DM, Waterman ML. A uniform human Wnt expression library reveals a shared secretory pathway and unique signaling activities. Differentiation. 2012;84(2):203–13. 10.1016/j.diff.2012.06.004 PMC4015730 22784633

[47] Fu J, Jiang M, Mirando AJ, Yu HM, Hsu W (2009). Reciprocal regulation of Wnt and Gpr177/mouse Wntless is required for embryonic axis formation. Proc Natl Acad Sci U S A.

[48] Maruyama T, Jiang M, Hsu W (2013). Gpr177, a novel locus for bone mineral density and osteoporosis, regulates osteogenesis and chondrogenesis in skeletal development. J Bone Miner Res.

[49] Rivadeneira F, Styrkarsdottir U, Estrada K, Halldorsson BV, Hsu YH, Richards JB, Zillikens MC, Kavvoura FK, Amin N, Aulchenko YS (2009). Twenty bone-mineral-density loci identified by large-scale meta-analysis of genome-wide association studies. Nat Genet.

[50] Zhang L, Choi HJ, Estrada K, Leo PJ, Li J, Pei YF, Zhang Y, Lin Y, Shen H, Liu YZ (2014). Multistage genome-wide association meta-analyses identified two new loci for bone mineral density. Hum Mol Genet.

[51] Zheng HF, Forgetta V, Hsu YH, Estrada K, Rosello-Diez A, Leo PJ, Dahia CL, Park-Min KH, Tobias JH, Kooperberg C et al. Whole-genome sequencing identifies EN1 as a determinant of bone density and fracture. Nature. 2015. 10.1038/nature14878 PMC4755714 26367794

[52] Roshandel D, Thomson W, Pye SR, Boonen S, Borghs H, Vanderschueren D, Huhtaniemi IT, Adams JE, Ward KA, Bartfai G (2011). Polymorphisms in genes involved in the NF-kappaB signalling pathway are associated with bone mineral density, geometry and turnover in men. PLoS One.

[53] Kiel DP, Demissie S, Dupuis J, Lunetta KL, Murabito JM, Karasik D (2007). Genome-wide association with bone mass and geometry in the Framingham Heart Study. BMC Med Genet.

[54] Richards JB, Kavvoura FK, Rivadeneira F, Styrkarsdottir U, Estrada K, Halldorsson BV, Hsu YH, Zillikens MC, Wilson SG, Mullin BH (2009). Collaborative meta-analysis: associations of 150 candidate genes with osteoporosis and osteoporotic fracture. Ann Intern Med.

[55] Cappello S, Gray MJ, Badouel C, Lange S, Einsiedler M, Srour M, Chitayat D, Hamdan FF, Jenkins ZA, Morgan T (2013). Mutations in genes encoding the cadherin receptor-ligand pair DCHS1 and FAT4 disrupt cerebral cortical development. Nat Genet.

[56] Mao Y, Mulvaney J, Zakaria S, Yu T, Morgan KM, Allen S, Basson MA, Francis-West P, Irvine KD. Characterization of a Dchs1 mutant mouse reveals requirements for Dchs1-Fat4 signaling during mammalian development. Development. 2011;138(5):947–57. 10.1242/dev.057166 PMC3035097 21303848

[57] Saburi S, Hester I, Fischer E, Pontoglio M, Eremina V, Gessler M, Quaggin SE, Harrison R, Mount R, McNeill H. Loss of Fat4 disrupts PCP signaling and oriented cell division and leads to cystic kidney disease. Nat Genet. 2008;40(8):1010–5. 10.1038/ng.179 18604206

[58] Fanjul-Fernandez M, Quesada V, Cabanillas R, Cadinanos J, Fontanil T, Obaya A, Ramsay AJ, Llorente JL, Astudillo A, Cal S (2013). Cell-cell adhesion genes CTNNA2 and CTNNA3 are tumour suppressors frequently mutated in laryngeal carcinomas. Nat Commun.

[59] Zheng HF, Duncan EL, Yerges-Armstrong LM, Eriksson J, Bergstrom U, Leo PJ, Leslie WD, Goltzman D, Blangero J, Hanley DA (2013). Meta-analysis of genome-wide studies identifies MEF2C SNPs associated with bone mineral density at forearm. J Med Genet.

[60] Cook SA, Bronson RT, Donahue LR, Ben-Arie N, Davisson MT (1997). Cerebellar deficient folia (cdf): a new mutation on mouse chromosome 6. Mamm Genome.

[61] Moayyeri A, Hsu YH, Karasik D, Estrada K, Xiao SM, Nielson C, Srikanth P, Giroux S, Wilson SG, Zheng HF (2014). Genetic determinants of heel bone properties: genome-wide association meta-analysis and replication in the GEFOS/GENOMOS consortium. Hum Mol Genet.

[62] Koller DL, Zheng HF, Karasik D, Yerges-Armstrong L, Liu CT, McGuigan F, Kemp JP, Giroux S, Lai D, Edenberg HJ (2013). Meta-analysis of genome-wide studies identifies WNT16 and ESR1 SNPs associated with bone mineral density in premenopausal women. J Bone Miner Res.

[63] Cheung CL, Sham PC, Xiao SM, Bow CH, Kung AW (2012). Meta-analysis of gene-based genome-wide association studies of bone mineral density in Chinese and European subjects. Osteoporos Int.

[64] Cauley JA (2015). Estrogen and bone health in men and women. Steroids.

[65] Vidal O, Lindberg M, Savendahl L, Lubahn DB, Ritzen EM, Gustafsson JA, Ohlsson C. Disproportional body growth in female estrogen receptor-alpha-inactivated mice. Biochem Biophys Res Commun. 1999;265(2):569–71. 10.1006/bbrc.1999.1711 10558910

[66] Heine PA, Taylor JA, Iwamoto GA, Lubahn DB, Cooke PS (2000). Increased adipose tissue in male and female estrogen receptor-alpha knockout mice. Proc Natl Acad Sci U S A.

[67] Smith EP, Boyd J, Frank GR, Takahashi H, Cohen RM, Specker B, Williams TC, Lubahn DB, Korach KS. Estrogen resistance caused by a mutation in the estrogen-receptor gene in a man. N Engl J Med. 1994;331(16):1056–61. 10.1056/NEJM199410203311604 8090165

[68] Smith EP, Specker B, Bachrach BE, Kimbro KS, Li XJ, Young MF, Fedarko NS, Abuzzahab MJ, Frank GR, Cohen RM (2008). Impact on bone of an estrogen receptor-alpha gene loss of function mutation. J Clin Endocrinol Metab.

[69] Pruim RJ, Welch RP, Sanna S, Teslovich TM, Chines PS, Gliedt TP, Boehnke M, Abecasis GR, Willer CJ. LocusZoom: regional visualization of genome-wide association scan results. Bioinformatics. 2010;26(18):2336–7. 10.1093/bioinformatics/btq419 PMC2935401 20634204

